# Essential waters: Young bull sharks in Fiji's largest riverine system

**DOI:** 10.1002/ece3.5304

**Published:** 2019-06-13

**Authors:** Kerstin B. J. Glaus, Juerg M. Brunnschweiler, Susanna Piovano, Gauthier Mescam, Franziska Genter, Pascal Fluekiger, Ciro Rico

**Affiliations:** ^1^ Faculty of Science, Technology and Environment, School of Marine Studies The University of the South Pacific Suva Fiji; ^2^ Independent Researcher Zurich Switzerland; ^3^ Projects Abroad Shark Conservation Project Fiji Goring‐by‐Sea UK; ^4^ Department of Environmental Systems Science ETH Zurich Zurich Switzerland; ^5^ Instituto de Ciencias Marinas de Andalucía (ICMAN) Consejo Superior de Investigaciones Científicas Puerto Real Cádiz Spain

**Keywords:** *Carcharhinus leucas*, essential fish habitats, neonates, salinity, South Pacific

## Abstract

Coastal and estuarine systems provide critical shark habitats due to their relatively high productivity and shallow, protected waters. The young (neonates, young‐of‐the‐year, and juveniles) of many coastal shark species occupy a diverse range of habitats and areas where they experience environmental variability, including acute and seasonal shifts in local salinities and temperatures. Although the location and functioning of essential shark habitats has been a focus in recent shark research, there is a paucity of data from the South Pacific. In this study, we document the temporal and spatial distribution, age class composition, and environmental parameters of young bull sharks (*Carcharhinus leucas*) in the Rewa, Sigatoka, and Navua Rivers, Fiji's three largest riverine systems. One hundred and seventy‐two young bull sharks were captured in fisheries‐independent surveys from January 2016 to April 2018. The vast majority of the captures were neonates. Seasonality in patterns of occurrence of neonate individuals suggests a defined parturition period during summer. Environmental parameters between the Rewa and the Sigatoka River differed significantly, as did the recorded young bull sharks abundance. According to the surveys, young bull sharks occur in all three rivers with the Rewa River likely representing essential habitat for newly born bull sharks. These results enhance the understanding of bull shark ecology in Fiji and provide a scientific basis for the implementation of local conservation strategies that contribute to the protection of critical habitats.

## 1 INTRODUCTION

Essential fish habitats (EFH) are “those waters and substrate necessary to fish for spawning, breeding, feeding or growing to maturity” (Rosenberg, Bigford, Leathery, Hill, & Bickers, 2000). Due to their relatively high productivity and shallow, protected waters, coastal and estuarine systems provide EFH for many continental shelf‐associated teleosts and elasmobranchs (Beck et al., 2001). For sharks, nursery areas (which are characteristically the most productive and consistent juvenile habitats over time (Heupel, Carlson, & Simpfendorfer, 2007)) are known for several members of the Carcharhinidae and Sphyrnidae families (Duncan & Holland, 2006; McCandless, Kohler, & Pratt, 2007; Yeiser, Heupel, & Simpfendorfer, 2008). However, even within the same species, such habitats can differ between and across regions, alter due to changing environments (Bangley, Paramore, Shiffman, & Rulifson, 2018), and may shift with the requirements of different size classes (Grubbs, 2010). Given the extinction risk many large‐bodied, shallow‐water species are facing (Dulvy et al., 2014), it is of importance to identify and characterize EFH both in space and time.

**Figure 1:**
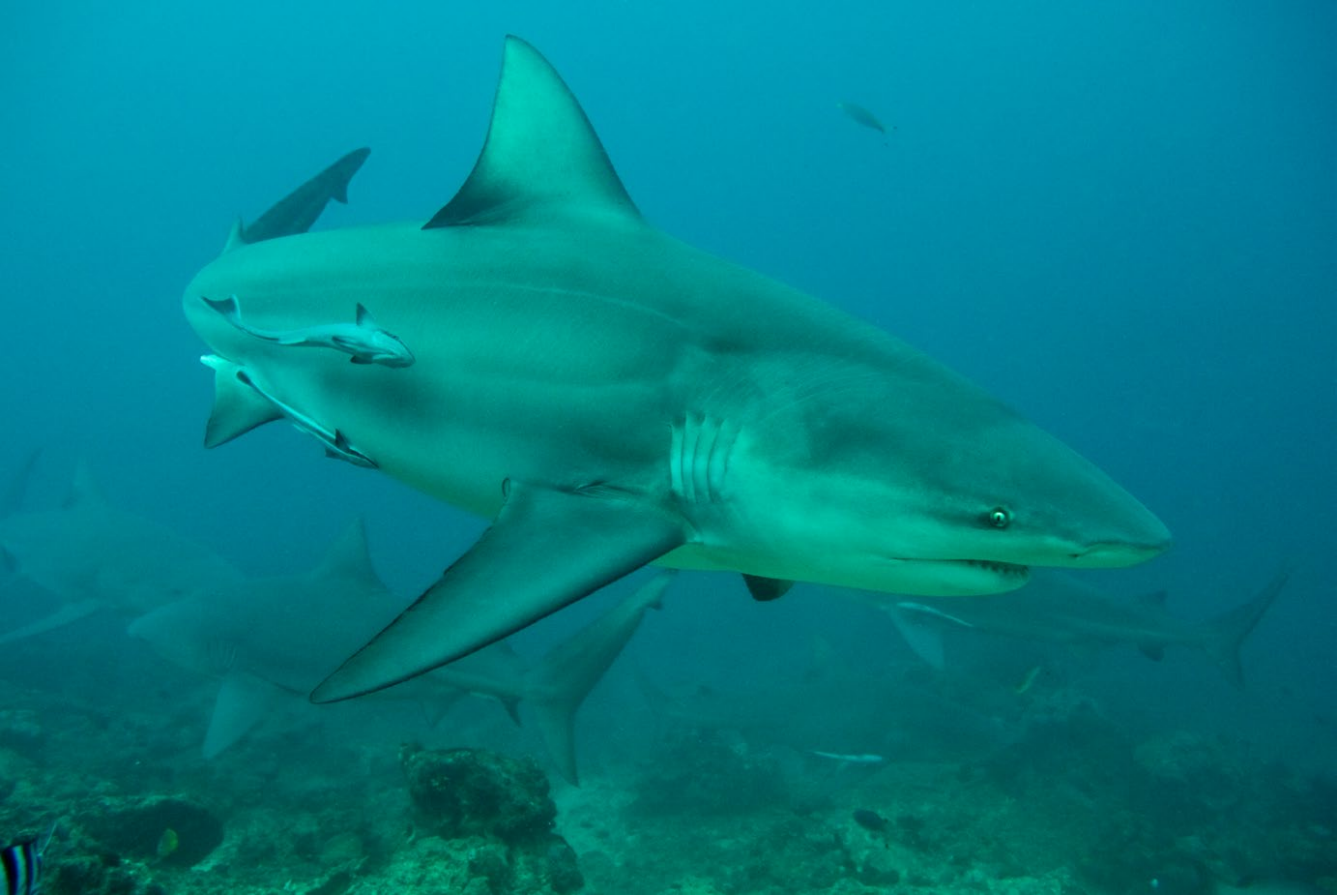
A bull shark (*Carcharhinus leucas*) photographed in Fiji's Shark Reef Marine Reserve. Copyright Valerie Taylor

The bull shark (*Carcharhinus leucas;* Figure 1) is considered as “Near Threatened” by the International Union for Conservation of Nature in the latest Shark Specialist Group assessment (Simpfendorfer & Burgess, 2007). The bull shark is a large coastal apex predator circumglobally distributed in tropical and warm temperate waters (Compagno, Dando, & Fowler, 2005). This euryhaline species has been reported from numerous freshwater systems within its global distribution range (Bass, D'aubrey, & Kistnasamy, 1975; Carlson, Ribera, Conrath, Heupel, & Burgess, 2010; Curtis, Adams, & Burgess, 2011; Daly, Smale, Cowley, & Froneman, 2014; Montoya & Thorson, 1982) and is well known to use shallow coastal regions and rivers as parturition sites and nursery grounds (Froeschke, Stunz, & Wildhaber, 2010; Heithaus, Delius, Wirsing, & Dunphy‐Daly, 2009). Neonate, young‐of‐the‐year (YOY), and juvenile bull sharks reportedly occupy environmentally heterogeneous habitats (Yates, Heupel, Tobin, & Simpfendorfer, 2012). For example, within subtropical regions, age‐associated habitat transitions have been documented with YOY bull sharks occupying locations with lower mean salinities than juveniles (Heithaus et al., 2009; Simpfendorfer, Freitas, Wiley, & Heupel, 2005), while subadults and adults were more abundant in nearshore marine areas (Werry, 2010; Werry, Lee, Otway, Hu, & Sumpton, 2011). These ontogenetic habitat shifts might be a successful ecological strategy for reducing juvenile mortality due to predator avoidance and as a result of changes in intra‐ and interspecific competition (Heithaus, 2004; Heupel & Simpfendorfer, 2011). Within coastal environments, neonate and YOY bull sharks experience environmental variability including acute and seasonal shifts in local salinities and water temperatures which can expose them to a range from 0 to 40 Practical Salinity Units (PSU) and 14.4–32.4°C, respectively (Froeschke et al., 2010; Heupel & Simpfendorfer, 2008). It is generally assumed that, because the bull shark is a euryhaline species (Pillans et al., 2006; Reilly, Cramp, Wilson, Campbell, & Franklin, 2011) that can respond to sudden changes in salinity with minimal metabolic costs (Anderson et al., 2006), salinity would not be an important factor influencing the species' distribution and habitat use patterns. However, juvenile bull sharks occur mostly in low to moderate salinities ranging from 10 to 30 PSU, rarely in salinities greater than 35 PSU (Froeschke et al., 2010), and may have an affinity for areas with salinities between 7 and 20 PSU (Matich et al., 2017; Simpfendorfer et al., 2005). Recent evidence suggests that rising water temperatures and increasing salinities can lead to expansions of the species' nursery areas toward higher latitudes (Bangley et al., 2018). To date, bull shark EFH have been identified and characterized primarily in the northern Gulf of Mexico, in Florida and on the east coast of Australia (Blackburn, Neer, & Thompson, 2007; Curtis et al., 2011; Heupel & Simpfendorfer, 2011), whereas information on bull shark EFH is largely lacking from areas elsewhere and in particular data‐poor regions such as the South Pacific.

The Republic of Fiji is an archipelago located in the South Pacific Ocean. At least 30 species of sharks including bull sharks are found in Fijian waters, many of which are resident species that probably spend all or much of their lives within Fiji's exclusive economic zone (Mangubhai et al., 2019). To date, information on parturition sites and nursery areas is known for only a few species. Marie, Miller, Cawich, Piovano, and Rico (2017) confirmed the Rewa Delta as important habitat for juvenile scalloped hammerhead sharks (*Sphyrna lewini*), and Vierus et al. (2018) discovered a multispecies shark aggregation and parturition area in the Ba Estuary on the northern coast of Viti Levu. The latter study documented three juvenile bull sharks caught several kilometers upstream in the Ba River. Juvenile bull sharks were also confirmed in the Navua River close to the Shark Reef Marine Reserve (SRMR) where large adult bull sharks are abundant (Brunnschweiler, Abrantes, & Barnett, 2014; Cardeñosa, Glaus, & Brunnschweiler, 2017). In addition to these observed occurrences, results from an interview‐based survey documented small sharks in all of Fiji's major rivers (Rasalato, Maginnity, & Brunnschweiler, 2010) suggesting that in particular Fiji's largest riverine systems, the Rewa and Sigatoka Rivers on the southern coast of Viti Levu represent EFH for young bull sharks.

In this study, our aims were to (a) confirm the occurrence of young bull sharks in the Rewa and Sigatoka Rivers, (b) determine their distribution and abundance in the rivers, and (c) collect environmental parameters at capture sites. We also include fishery‐dependent data on the size, umbilical scar condition, and sex of young bull sharks captured opportunistically by local fishermen in the Navua River.

## 2 MATERIAL AND METHODS

### 2.1 Interviews and identification of sampling sites

To identify sampling sites, 35 fishermen from seven different villages along the Rewa River were interviewed in February 2016. In addition, interviews were conducted with representatives from the Ministry of Fisheries and Forests in Nausori Town and in Wainibokasi situated along the Rewa River, and in Sigatoka Town. Information was gathered by means of questionnaire‐based interviews following the methods described in Glaus, Adrian‐Kalchhauser, Burkhardt‐Holm, White, and Brunnschweiler (2015). In brief, as per village protocol, permission was requested from village chiefs to interview fishermen in their respective villages. Chiefs would then designate participants. All interviews were conducted by one of the authors (K.G.), who was accompanied by two Fijian collaborators. Fijian collaborators were fluent both in English and Fijian Bauan dialect. Each interview started with an explanation about the main purpose of the survey. Names of participants were not noted to guarantee their anonymity. Fishermen were interviewed individually and asked whether they observe sharks in the respective river. If yes, participants were further asked to provide information on the spatial and temporal distribution, body shape, color, and approximate size of the sharks they observe. Subsequently, sampling sites within the Rewa and Sigatoka Rivers (see below in the fishery‐independent surveys) were chosen following fishermen's local ecological knowledge (Rasalato et al., 2010) who suggested areas where they previously caught sharks and upon recommendations by the Ministry of Fisheries and Forests. The sampling scheme in the Navua River was similar to the one applied in Cardeñosa et al. (2017).

### 2.2 Fishery‐independent surveys

**Figure 2:**
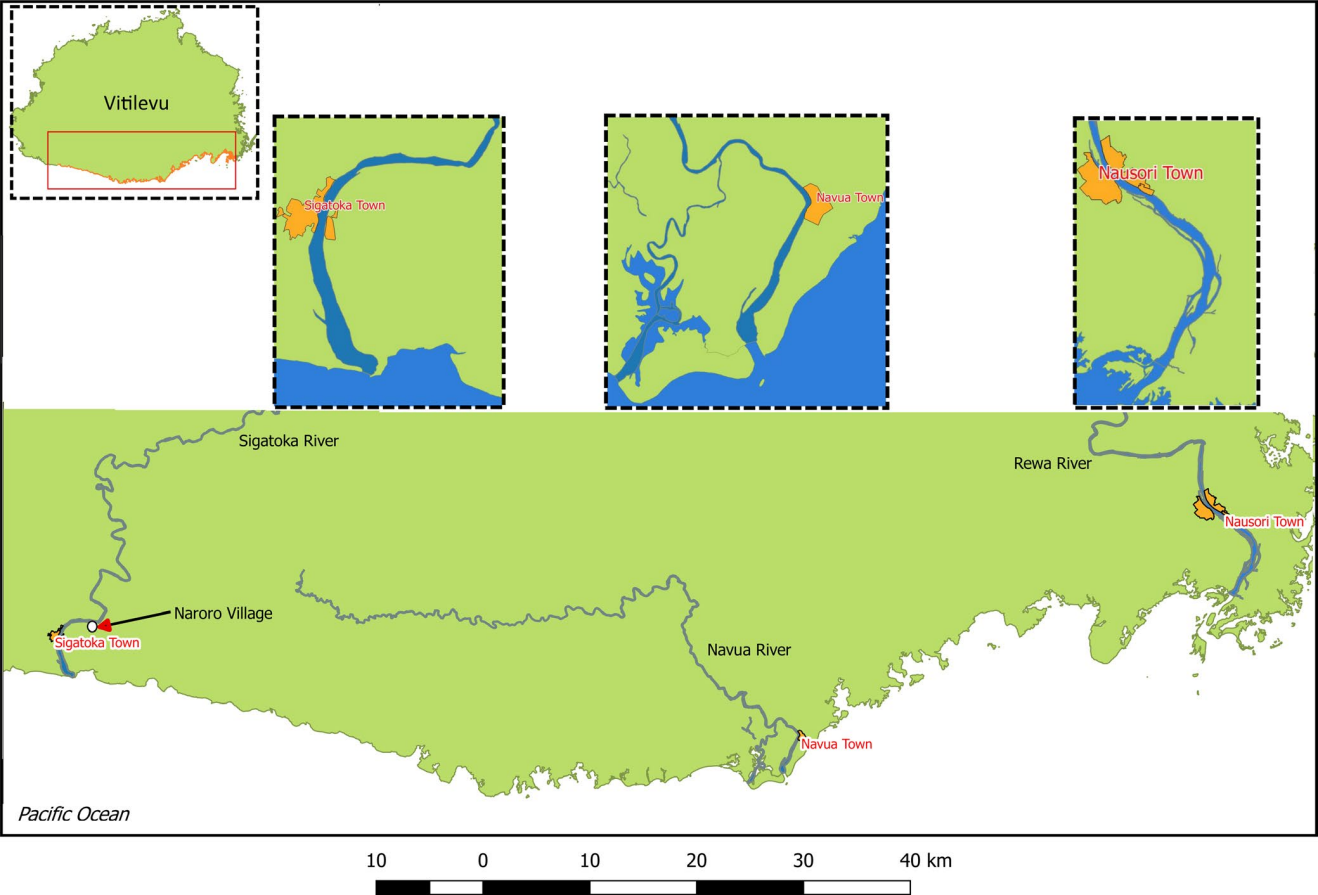
The Rewa, Sigatoka, and Navua Rivers in southern Viti Levu. Dashed inlets denote the stretches that were sampled

To assess the occurrence and abundance of neonate, YOY, and juvenile bull sharks in Fiji's three largest riverine systems on the southern coast of Viti Levu, vessel‐based fisheries‐independent surveys were conducted in the Rewa, Sigatoka, and Navua Rivers between 2016 and 2018, spanning over two parturition seasons (Brunnschweiler & Baensch, 2011). Survey periods and sampling hours are summarized in Table 1. Lower to mid reaches and estuaries were surveyed; sampling sites in the Rewa, Sigatoka, and Navua Rivers were from the river mouth to Nausori Town 15 km upstream, Naroro Village 7.7 km upstream, and from Navua Town 3.5 km upstream, respectively (Figure 2). The Navua River has a tributary, the Deuba River, which, if not otherwise indicated, was included in the Navua River.

**Table 1:** Summary of the sampling effort in each river surveyed

River	Survey period	Sampling hours 2016/2017	Survey period	Sampling hours 2017/2018
Rewa	March 2016 to March 2017	322	November 2017 to April 2018	99
Sigatoka	October to December 2016	191	October 2017 to February 2018	196
Navua	January to December 2016	384	October 2017 to February 2018	135

As tidal states have been linked to shark presence and movements (Grubbs, Musick, Conrath, & Romine, 2007; Heupel et al., 2007), surveys started at low tide and typically lasted between 2 and 6 hr per day depending on weather conditions. Sites were sampled with a gillnet (150 × 3 m) made of 4‐inch and 9‐ply mesh. Deployed gillnets were inspected every 20–35 min to reduce the risk of animal casualties. Bull sharks that were caught in the mesh were placed in an on‐board tank filled with river water. The following parameters were recorded for each specimen caught: total straight length (TL), umbilical scar condition (open, semihealed, healed, Duncan & Holland, 2006), and sex. Bull sharks were not taken on‐board but released immediately if signs of physiological stress responses (e.g., red skin) or lesions were visible. All captured bull sharks were tagged with an internal Passive Integrated Transponder (Animal ID ISO‐Compliant‐Transponder RFID Microchip tag) below the first dorsal fin for individual identification. In addition, using a YSI‐85 water quality meter, surface and bottom water temperature, dissolved oxygen (DO), and salinity were recorded at the respective sampling locations in the Rewa and Sigatoka Rivers at the beginning and end of each fishing survey.

Here, sampling effort refers to the standardized amount of time spent sampling with a standard fishing gear in hours and days. For the Rewa and Sigatoka Rivers, sampling effort was calculated as total number of hours sampled in a month divided by the number of days of the respective month multiplied by the total number of days actually sampled in that month. The Navua River was surveyed on 27 hr per month on average as part of the Project's Abroad Shark Conservation project in Fiji. Catch per unit of effort (CPUE) was standardized by summing the total number of bull sharks caught and divided by 150 m (length) × 3 m (height) of the net over 1‐hr time period for each net set. Biological data from the bull sharks were visualized using R Studio 3.4.0 (R Core Team, 2016) and included both fishery‐dependent and fishery‐independent data.

### 2.3 Fishery‐dependent data

Fishery‐dependent data were obtained from two local fishermen from the Navua River between January 2016 and May 2017. One of the authors (G.M.) knew the fishers personally, and they were asked to make contact when they caught a bull shark. The fishermen were informed about the purpose of the survey and were encouraged to release caught sharks if alive. No financial incentives were given in return for bull shark carcasses.

### 2.4 Data analysis

Surface and bottom values of each environmental parameter were averaged, and to test whether the environmental parameters in the Rewa and Sigatoka Rivers had equal means, a Welch's *t* test was performed in R version 3.5.0. For modeling bull shark occurrence in relation to environmental conditions and the river system, we calculated a full binomial generalized linear model (GLM) with a logistic link function. The model contained environmental parameters for deployment (temperature, salinity, and dissolved oxygen), a dummy variable for river (1: Rewa, 0: Sigatoka), and the log transformed sampling time for each deployment. In the model, we included river explicitly as a fixed rather than a random effect, because we intended to estimate the effect of river in a model where other environmental parameters are accounted for. The response variable was binary and equals one when a deployment led to a catch and zero otherwise. All subsets of this model were calculated, holding log_2 _(time of sampling) fixed as a control variable in all models. The two best‐fit models were selected via Akaike's information criterion (AIC; Bozdogan, 1987), according to the criterion delta AIC < 2. These models were averaged using the R package *MuMIn* (Barton & Barton, 2018).

## 3 RESULTS

The presence of young sharks in the Rewa, Sigatoka, and Navua Rivers was confirmed by both the interviews with local fishers and the fishing surveys. In total, 172 bull sharks were caught during the fishery‐independent surveys in the three rivers over the course of 2 years, and 22 specimens by fishery‐dependent surveys in the Navua River. The number of sharks caught per day in the Rewa River ranged from one to 22 individuals.

### 3.1 Bull shark occurrence and catch rates

**Figure 3:**
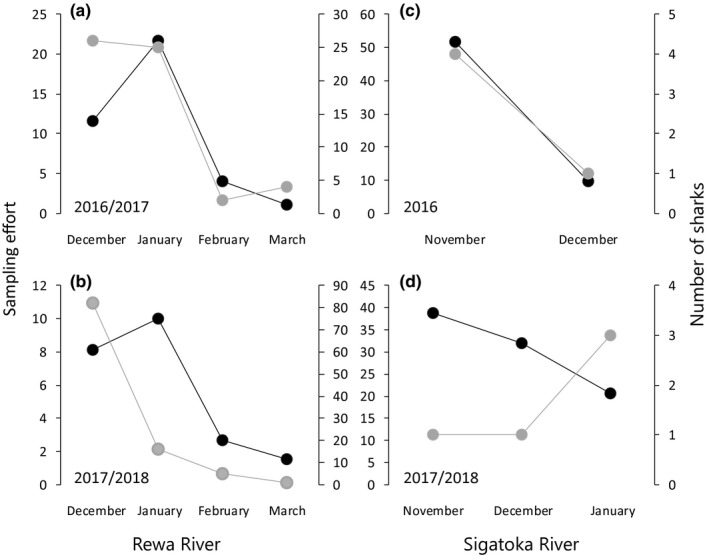
Standardized sampling effort (dark) and number of sharks captured (light) in the Rewa and Sigatoka Rivers in the 2016/2017 survey period (a and c), and the 2017/2018 survey period (b and d)

**Figure 4:**
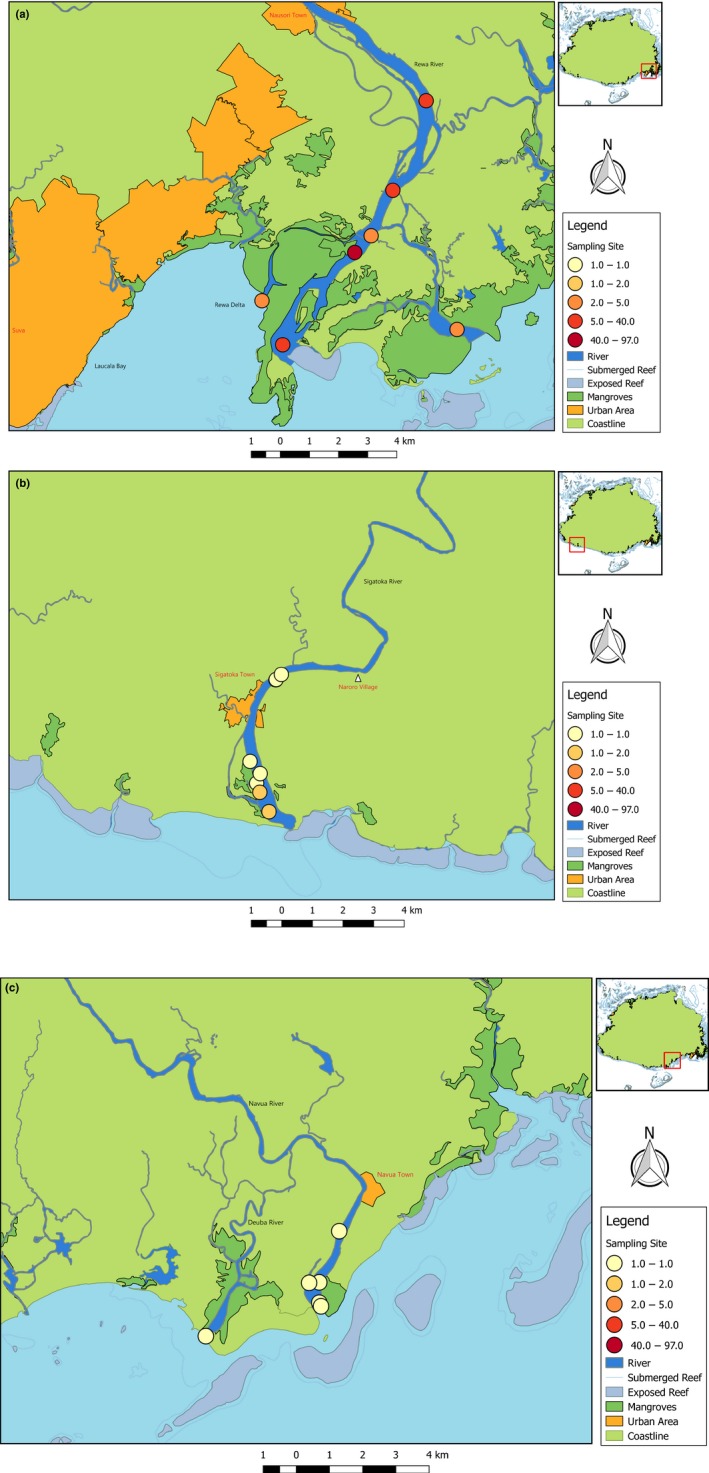
Capture sites within the (a) Rewa, (b) Sigatoka, and (c) Navua Rivers. Color of the circles indicates the number of bull sharks caught at the respective site with red circles denoting sites where large numbers of bull sharks were captured

In the Rewa River, no bull sharks were caught between March and November 2016 (192 sampling hours). Between December 2016 and March 2017, 57 individual bull sharks were caught at seven sampling sites from the estuaries to 8.5 km upstream from the river mouth (Figure 3a). Most bull sharks (*n* = 26) were caught in December 2016; a similar number (*n* = 25) was caught in January 2017 despite almost double sampling effort (Figure 4a). Between December 2017 and March 2018, 104 bull sharks were caught at three sampling sites within the lower reaches of the Rewa River (Figure 3a). These three sampling sites were also sampled during the previous survey in 2016/2017. No sharks were caught in the months of November 2017 and April 2018 (20 sampling hours). Again, most bull sharks (*n* = 82) were caught in December 2017, but in this survey period, a much lower number (*n* = 16) was caught in January 2018 despite a similar sampling effort (Figure 4b). Two specimens were recaptured in the 2017/2018 survey period at the same sampling site where they were tagged 59 and 74 days earlier, respectively. Pooled CPUEs within the Rewa River ranged between 0 and 12.5 sharks/hr (Table 2), with the highest monthly CPUE recorded in December 2017, while surveys in February had the lowest CPUEs (0.7–1 sharks/hr) both in 2017 and 2018 (Figure 4a,b).

**Table 2:** Sampling effort and average CPUE per river and survey period

River	Survey period	Sampling effort (hr)	Mean CPUE [range]
Rewa	March to November 2016	192	0
Rewa	November 2016 to March 2017	130	0.438 [0–5]
Rewa	November 2017 to March 2018	99	1.05 [0–12.5]
Sigatoka	October to December 2016	191	0.026 [0–2]
Sigatoka	October 2017 to February 2018	196	0.025 [0–2]
Navua	January 2016 to December 2016	384	0.005 [0–1]
Navua	October 2017 to February 2018	135	0

In the Sigatoka River, despite a considerable sampling effort (Tables 1 and 2), only five bull sharks were caught in each survey period (Figure 4c,d). These 10 bull sharks were caught at seven sampling sites as far as 5.3 km upstream from the river mouth (Figure 3b). No specimens were recaptured. Pooled CPUEs within the Sigatoka River ranged between 0 and 2 sharks/hr (Table 2).

Similar to the Sigatoka River and despite a relatively high sampling effort, only two bull sharks were caught during fishery‐independent surveys in the Navua River in 2016, one each in June and in July. The individual caught in July was a recapture after 131 days at liberty near the river mouth of the Deuba River (Figure 3c). In total, the two fishers from the Navua River reported 14 individual bull sharks caught between January and November 2016 and eight bull sharks caught between January and May 2017.

### 3.2 Biological data and population structure

**Figure 5:**
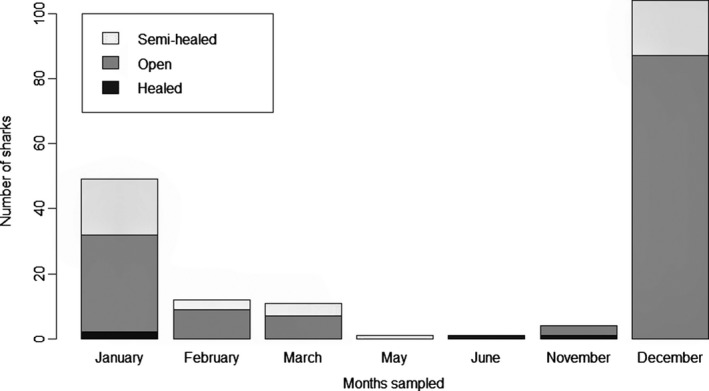
Umbilical scar conditions of bull sharks captured in the Rewa, Sigatoka, and Navua Rivers indicated per month across the whole study duration. Recaptured individuals are excluded

In total, 194 individual specimens were sexed, their umbilical scar condition was recorded, and all but one were measured. Of these, 99 (51%) were males and 95 (49%) were females. Open umbilical scars were detected in 145 individuals (74.7%), 45 individuals (23.2%) were classified as semihealed, and the umbilical scars in four individuals (2.1%) were healed (Figure 5). The majority of the individuals caught between December and March had an open umbilical scar. Fully healed scars were encountered in two individuals caught in the Sigatoka River and in two caught in the Navua River. None of these specimens was a recaptured individual.

**Figure 6:**
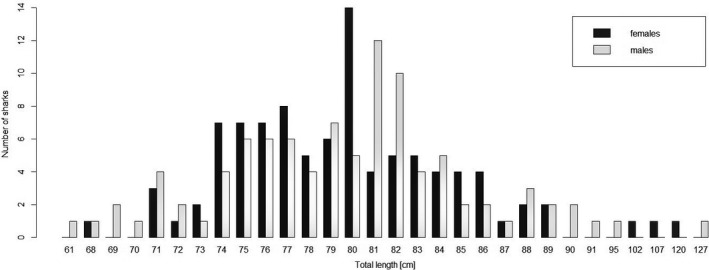
Total length distributions of bull sharks captured in the Rewa, Sigatoka, and Navua Rivers from January 2016 to April 2018. Total length and corresponding number of males and females are represented in white and black, respectively. Recaptured individuals are excluded

Based on individual TL measurements, the presence of at least three age classes (i.e., neonates, YOY, and 1+ year) was inferred (Figure 6). Bull sharks ranged from 61 to 127 cm TL (Figure 6). The recaptured individual in the Navua River grew 11 cm in TL after 131 days at liberty. The two bull sharks recaptured in the Rewa River after 59 and 74 days each grew 4 cm in TL. Growth in these recaptured individuals translated to average of 1.6, 2, and 2.5 cm per month.

### 3.3 Environmental parameters Rewa versus Sigatoka River

**Figure 7:**
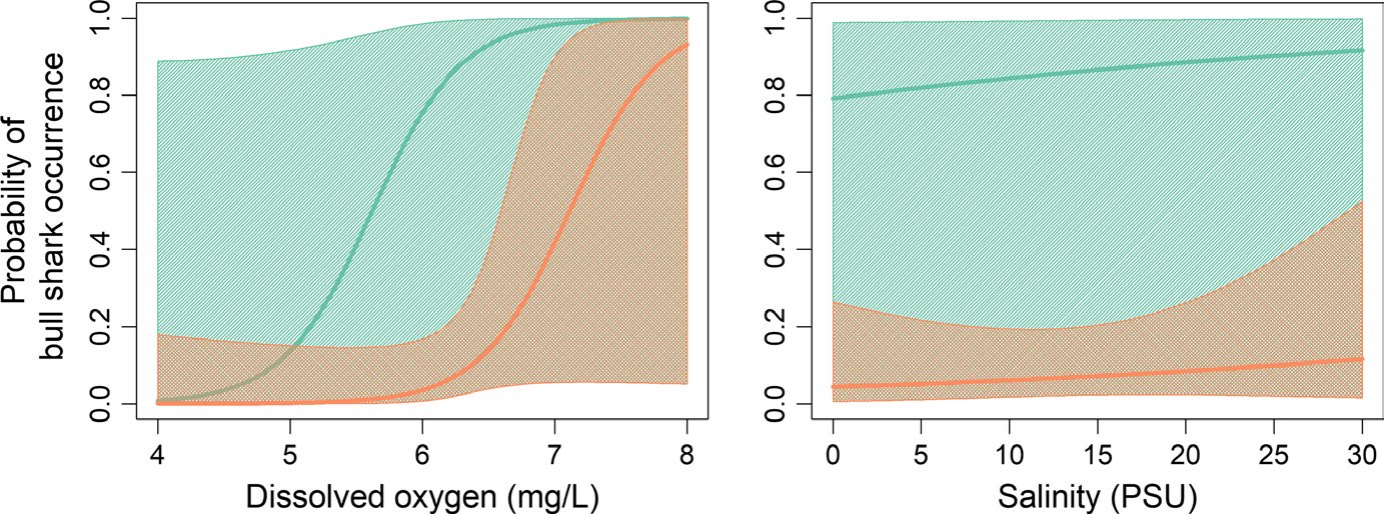
Predicted probability of capturing a bull shark for the Rewa River (green) and the Sigatoka River (orange) and environmental conditions (left panel: dissolved oxygen, right panel: salinity). Shaded areas represent 95% confidence intervals. Predictions are based on averaged model estimates (Table 4). All other parameters of the model not visualized in the respective plots are held at their empirical median

Salinity and DO statistically differed significantly between the Rewa and Sigatoka Rivers. Mean salinity highly differed between the two rivers. On average, DO concentration and water temperature in the sampled sites were higher in the Rewa than in the Sigatoka River (Table 3). The two best‐fit GLM models did not contain temperature as a predictor, and hence neither did the averaged model. Shark occurrence increased with salinity and dissolved oxygen, although, due to the small sample size (full environmental conditions were only available for *n* = 56 deployments), the uncertainty of these effects is high (Table 4, Figure 7). Despite accounting for these environmental parameters, there was still an effect of river: Sharks were more likely to occur in Rewa than in Sigatoka, which is also supported also by our descriptive statistics (Figure 4).

**Table 3:** Statistical differences based on Welch's *t* test between the environmental parameters measured in the two rivers

**Dissolved oxygen** (mg/L)		
*t* = 2.51	*df* = 10.48	*p*‐value = 0.03
Mean Rewa: 6.6 mgL^−1^	Mean Sigatoka: 6.1 mg/L	
**Salinity** (ppt)		
*t* = −6.24	*df* = 43.90	*p*‐value = <0.01
Mean Rewa: 1.2 ppt	Mean Sigatoka: 11.2 ppt	
**Temperature (°C)**		
*t* = −2.0	*df* = 11.86	*p*‐value = 0.06
Mean Rewa: 27.8°C	Mean Sigatoka: 28.8°C	

**Table 4:** Results from averaging of the two best‐fit GLMs (∆ AIC < 2)

Variable	Estimate [Confidence interval]
Rewa River	3.90 [0.56, 7.24]
Dissolved oxygen	3.26 [−0.40, 6.93]
Salinity	1.90 [−1.03, 4.83]
Log_2_(sampling time)	−0.62 [−3.85, 2.61]

## 4 DISCUSSION

This study represents the first multiyear investigation on the occurrence and abundance of young bull sharks in Fiji's three largest riverine systems. Our results confirm the presence of young bull sharks in all three surveyed rivers. The capture of 159 neonate and YOY individuals in the Rewa River over two parturition periods (Brunnschweiler & Baensch, 2011) indicates that the surveyed area provides EFH for this coastal shark species.

### 4.1 Bull shark occurrence

Essential shark habitats include both “nurseries” and “other young shark habitats” (Beck et al., 2001). Even though great attention has been given to identifying shark nurseries to guide the focus of management and conservation efforts, it is now well accepted that non‐nursery habitats also contribute significantly to the adult population (Dahlgren et al., 2006; Yates et al., 2012). It is important to note that the comparison of multiple areas in terms of their contributions to adult stocks is difficult to quantify. Furthermore, the degree of site‐fidelity and distance between essential young bull shark habitats may directly affect the level of population subdivision and genetic divergence among regions, as well as the associated population dynamics. Genetic studies are currently underway in Fiji aiming to address these questions (K.G. unpublished data).

The temporal occurrence of neonate bull sharks in the surveyed rivers is largely consistent with results of other studies (Matich et al., 2017). In the Rewa River however, the spatial occurrence of neonate specimens differs from data published elsewhere. For example, Heupel and Simpfendorfer (2011) reported that neonate bull sharks occurred in mesohaline estuaries, while in the present study the vast majority of neonate bull sharks were captured in oligohaline waters. Interestingly, our sampling effort focused on selected habitats within the river after extensive sampling of the estuaries and the Rewa Delta rarely resulted in bull shark captures (e.g., Marie et al., 2017).

### 4.2 Population structure and reproductive biology

In the three surveyed rivers, 74.7% of captured bull sharks had an open umbilical scar, likely indicating that these rivers serve as parturition grounds for bull sharks in Viti Levu. Neonate bull sharks were encountered continuously in the Rewa River from December to March, and we did not encounter healed umbilical scars or an increase in length over the study period. These results suggest that the parturition period in Fiji's southern riverine systems occurs during the wet austral summer season, with a possible peak between December and January. This is in line with Cardeñosa et al. (2017), who reported that bull shark sightings or catches by fishers in Fiji mainly occur during summer, and also largely overlap with the characterization of other shark species' parturition season in Fiji (Marie et al., 2017; Vierus et al., 2018). Furthermore, parturition occurring in the austral summer corresponds with direct observations made in the SRMR where pregnant bull sharks leave the feeding site in late October, returning after parturition at the beginning of the year (Brunnschweiler et al., 2014; Brunnschweiler & Baensch, 2011).

At birth, bull sharks are reportedly between 70 and 82 cm in stretch total length (Simpfendorfer et al., 2005). In this study, the size of specimens with an open umbilical scar ranged between 61 and 95 cm total length, with most individuals measuring from 75 to 85 cm. Thus, size ranges were slightly higher than previously documented in other studies and regions (Branstetter & Stiles, 1987; Curtis et al., 2011; Simpfendorfer et al., 2005). However, it is reasonable to assume that not all populations have the same size ranges and potential reasons for this result (including phenotypic and genotypic variation) are hypothetical and not tested here. Nevertheless, the observed size range provides some evidence that the coastal waters in Fiji and in particular the Rewa River do contain adequate prey volume to support its young bull shark populations.

Young bull sharks in this study were not prone to recapture; only three individuals were recaptured 59, 74, and 131 days after they were first tagged. Measured growth rates are lower than documented by Cardeñosa et al. (2017); however, the low number of recaptured sharks is insufficient to draw any conclusions. The rare captures of individuals with a healed umbilical scar, the low number of recaptured bull sharks, and the lack of captures between April and November suggest that the sampling areas (i.e., the rivers) are used by neonates but not frequently used by YOY and juvenile bull sharks. This does not match findings based on a 30‐year synthesis on bull shark occurrence in the Indian River Lagoon, Florida (Curtis et al., 2011), where the area was frequently used by Age‐0 and juvenile bull sharks. Given our large sampling effort over multiple areas, it is reasonable to assume that the capture of YOY and juvenile sharks would have been expected. Multiple reasons and combinations thereof can result in the observed lack of shark captures during the austral winter months, and potential hypotheses for the lack of YOY and juvenile bull sharks in the Rewa River range from impacts of mortality (natural and/or by fishing pressure; Glaus et al., 2015), detrimental effects on shark habitats, altering habitat use patterns, selective gear bias (Drymon, Ajemian, & Powers, 2014; Heithaus et al., 2009), and learning behavior toward fishing gear avoidance (Guttridge, Myrberg, Porcher, Sims, & Krause, 2009). In addition, the Sigatoka River was not surveyed during winter months and further research is required to evaluate shark distribution patterns during this period. The paucity of bull shark captures in the Navua River confirms the results of Cardeñosa et al. (2017), despite the larger sampling area and extended sampling period in the present study. On a broader scale, there was one specific event that occurred during the beginning of the present survey which is noteworthy for its potential impact on bull shark occurrence, movement, and activity in the study areas. The Category 5 tropical cyclone Winston made landfall in Fiji on the 20th of February 2016. Some shark species have been reported to leave their nursery area during the approach of a tropical storm (Heupel, Simpfendorfer, & Hueter, 2003). It is therefore plausible that the young bull sharks within the Rewa, Navua, and Sigatoka Rivers may have responded to this severe climatic event in some manner.

### 4.3 Environmental parameters

The abundance of young age‐classes of bull sharks is most often associated with temperatures greater than 20°C, salinities of 10–30 PSU or PSU, and DO concentrations between 4 and 7 mg/L (Curtis et al., 2011), with neonates occupying waters with 5–18 PSU (Heupel & Simpfendorfer, 2011). The environmental profile of the Sigatoka River mirrors these ranges in all aspects; however, the profile of the Rewa River does not. Areas with highest bull shark abundance in the Rewa River typically were oligohaline (mean 1.2 PSU). Bull sharks can osmoregulate over the full range of salinity from freshwater to saltwater. Salinity preference is thought to minimize the energy required for osmoregulation and might represent an optimal condition for growth (Heupel & Simpfendorfer, 2008). Given that neonate bull sharks in the Rewa River had larger sizes at birth, this could indicate that they allocate energy for osmoregulation rather than for growth. Although the results from the present study were from a single potential EFH, the consistent oligohaline conditions in which neonate bull sharks occurred expand previous findings on preferred salinity ranges. Habitat selection patterns are, especially for young sharks, not well understood. Our modeling results suggested that bull shark occurrence increased with salinity and dissolved oxygen, and that they are more likely to occur in the Rewa than in the Sigatoka River. However, small sample sizes led to a high uncertainty of these effects. Although there is a difference in the rivers, this difference was likely not captured by the here measured parameters. Young bull sharks are able to readily adapt to urbanized estuaries and rivers (Heupel & Simpfendorfer, 2011), but different habitats may be differentially impacted by anthropogenic disturbance or environmental change. For example, in an interview‐based survey, fishermen along the Sigatoka River consistently stated that while small and large bull sharks were sighted regularly in this river 10–15 years ago, they are rare nowadays (Glaus et al., 2018). Weather conditions, dredging, and mining activities possibly altered the river's physical nature, which may not permit occupancy by sharks anymore. Contrastingly, dredging activities in the lower parts of the Rewa River were not conducted as the Rewa Delta designates a critical habitat for the endangered scalloped hammerhead shark (see Marie et al., 2017). In addition, other environmental parameters, biological factors (i.e., prey availability), or a combination of both may lead to the observed differentiation in bull shark occurrence between the Rewa and Sigatoka River, a pattern that definitely requires additional scrutiny, including higher number of sample sizes. Passive acoustic telemetry and the availability of additional site‐specific data such as prey abundance or oceanographic measurements could help to further examine the species' habitat use patterns and identify important drivers of their presence within and outside the rivers.

An increasingly clear picture of shark species distribution and abundance throughout Fiji is emerging (Cardeñosa et al., 2017; Marie et al., 2017; Piovano & Gilman, 2017; Vierus et al., 2018). Our multiple‐year assessment of three rivers in Fiji provides a first attempt at delineating essential habitats and at the identification of environmental parameters likely shaping neonate bull shark's distribution patterns. Improving our ability to manage coastal shark stocks is critical as shark populations have declined in the South Pacific Ocean (Clarke, Harley, Hoyle, & Rice, 2013). The development of spatially explicit models would allow for prioritization of areas for conservation and could provide insights into critical ecosystem attributes (i.e., salinity regimes) that merit protection. Given Fiji's voluntary commitment during the 2017 United Nations Ocean Conference to the conservation of all elasmobranchs within its territorial waters by 2020, the most promising management approach to protect young age‐classes of different shark species in Fiji is to give essential habitats a protected area status. This would include core zones where fishing is not allowed and enforcement of the existing gillnet ban in Fiji's rivers and estuaries.

## CONFLICT OF INTEREST

None declared.

## AUTHOR CONTRIBUTIONS

K.G., J.M.B., and C.R. designed the study. C.R. and S.P. wrote the project proposal. C.R., S.P., and K.G. obtained funding. K.G., G.M., F.G., and P.F. conducted the fieldwork. K.G., J.M.B., and G.M. developed the sampling scheme. K.G., J.M.B., and F.G. did the analyses. K.G. and J.M.B. wrote the first draft of the manuscript. All authors contributed to the write up of the final version of the manuscript. This study is part of the PhD‐thesis of K.G., supervised by C.R., S.P., and J.M.B.

## ETHICAL APPROVAL

Sampling was conducted under a research permit issued by Fiji Immigration Department to K.G., F.G., P.F., and G.M. Research permits for K.G., F.G., and P.F. were approved by the University of the South Pacific and the Secretary of Education. Additionally, a research permit was provided by Fiji Department of Fisheries. All handling procedures of live shark specimens were approved under the “Animal Ethics Committee” section of the USP Research Committee and performed in accordance with relevant guidelines and regulations.

## Data Availability

Data are permanently archived at the Dryad Digital Respiratory, https://datadryad.org DOI: https://doi.org/10.5061/dryad.47h01t4.
